# Strategic Inferior Mesenteric Artery Revascularization for an Inferior Mesenteric Artery Aneurysm Associated with Chronic Occlusion of the Celiac and Superior Mesenteric Arteries

**DOI:** 10.70352/scrj.cr.26-0499

**Published:** 2026-09-17

**Authors:** Shuhei Miura, Hajime Maeda, Takayuki Magishi, Shingo Tsushima, Atsuhiro Koya, Masami Shingaki

**Affiliations:** Department of Cardiovascular Surgery, Hakodate Municipal Hospital, Hakodate, Hokkaido, Japan

**Keywords:** inferior mesenteric artery aneurysm, mesenteric occlusive disease, arc of Riolan, revascularization

## Abstract

**INTRODUCTION:**

Inferior mesenteric artery (IMA) aneurysms are extremely rare visceral artery aneurysms and may develop secondary to chronic mesenteric occlusive disease. The optimal revascularization strategy remains controversial when both the celiac artery (CA) and superior mesenteric artery (SMA) are occluded.

**CASE PRESENTATION:**

A 75-year-old man with a 3.0-cm IMA aneurysm associated with chronic total occlusion of both the CA and SMA due to severe aortic calcification was referred to our institution. To minimize the risk of mesenteric ischemia during planned aortic surgery, a staged approach was adopted. Open repair was performed using a retrograde bypass from the left external iliac artery to the IMA with an 8-mm expanded polytetrafluoroethylene graft, followed by complete aneurysm resection. SMA revascularization was not performed because extensive calcification precluded endovascular intervention and alternative bypass procedures would have increased surgical complexity and invasiveness. Postoperative imaging demonstrated graft patency and preserved mesenteric perfusion through the arc of Riolan.

**CONCLUSIONS:**

Strategic isolated IMA revascularization may be a feasible option in selected patients with IMA aneurysms associated with chronic mesenteric occlusive disease when SMA revascularization is anatomically unsuitable. Long-term surveillance remains essential.

## INTRODUCTION

An inferior mesenteric artery (IMA) aneurysm is exceedingly rare, accounting for less than 1% of all visceral artery aneurysms.^1)^ Reported etiologies include degenerative or inflammatory disease, fibromuscular dysplasia, connective tissue disorders, and inherited vascular conditions.^2)^ Another proposed etiology is increased collateral blood flow secondary to occlusive disease of other mesenteric arteries.^3–5)^ This high-flow state may cause turbulence, increased wall stress, and progressive arterial dilatation, ultimately resulting in aneurysm formation.^3,6)^ We describe a 75-year-old man with a 3.0-cm IMA aneurysm in the setting of chronic total occlusion (CTO) of both the celiac artery (CA) and superior mesenteric artery (SMA), highlighting the pathophysiology and surgical strategy for this rare condition.

## CASE PRESENTATION

**Fig. 1:**
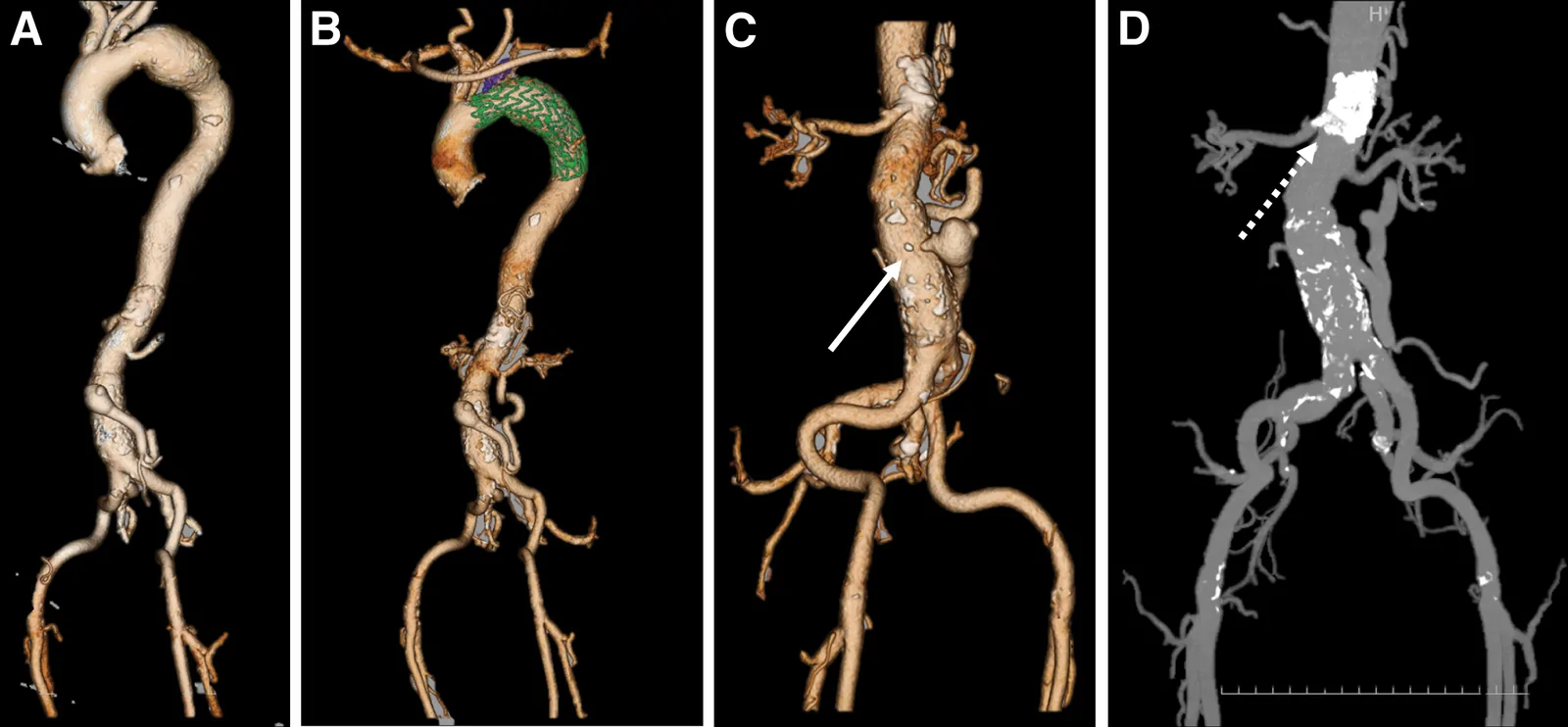
(**A**) 3D CTA revealed a TAA measuring 5.6 cm, an IMA aneurysm measuring 3.0 cm, and CTO of the CA and SMA. (**B**) Debranching TEVAR was performed as the first-stage procedure for the TAA. (**C**) 3D CTA viewed from a slight right anterior oblique angle demonstrates the spatial relationship between the saccular IMA aneurysm and the abdominal aorta and the absence of an adequate clamping zone at the IMA orifice (arrow). (**D**) Extensive aortic calcification obstructing the orifices of the CA and SMA (dotted arrow). CA, celiac artery; CTO, chronic total occlusion; IMA, inferior mesenteric artery; SMA, superior mesenteric artery; TAA, thoracic aortic aneurysm; TEVAR, thoracic endovascular aortic repair

A 75-year-old man with moderate aortic regurgitation and a 5.6-cm thoracic aortic aneurysm was referred to our institution. He was a current smoker with a history of hypertension. Screening CT revealed a 3.0-cm saccular IMA aneurysm arising from the proximal IMA. Extensive aortic calcification had resulted in occlusion of the CA and SMA ostia, with collateral perfusion of the distal SMA through the arc of Riolan (**Fig. 1A**, **1C**, **1D**).

**Fig. 2:**
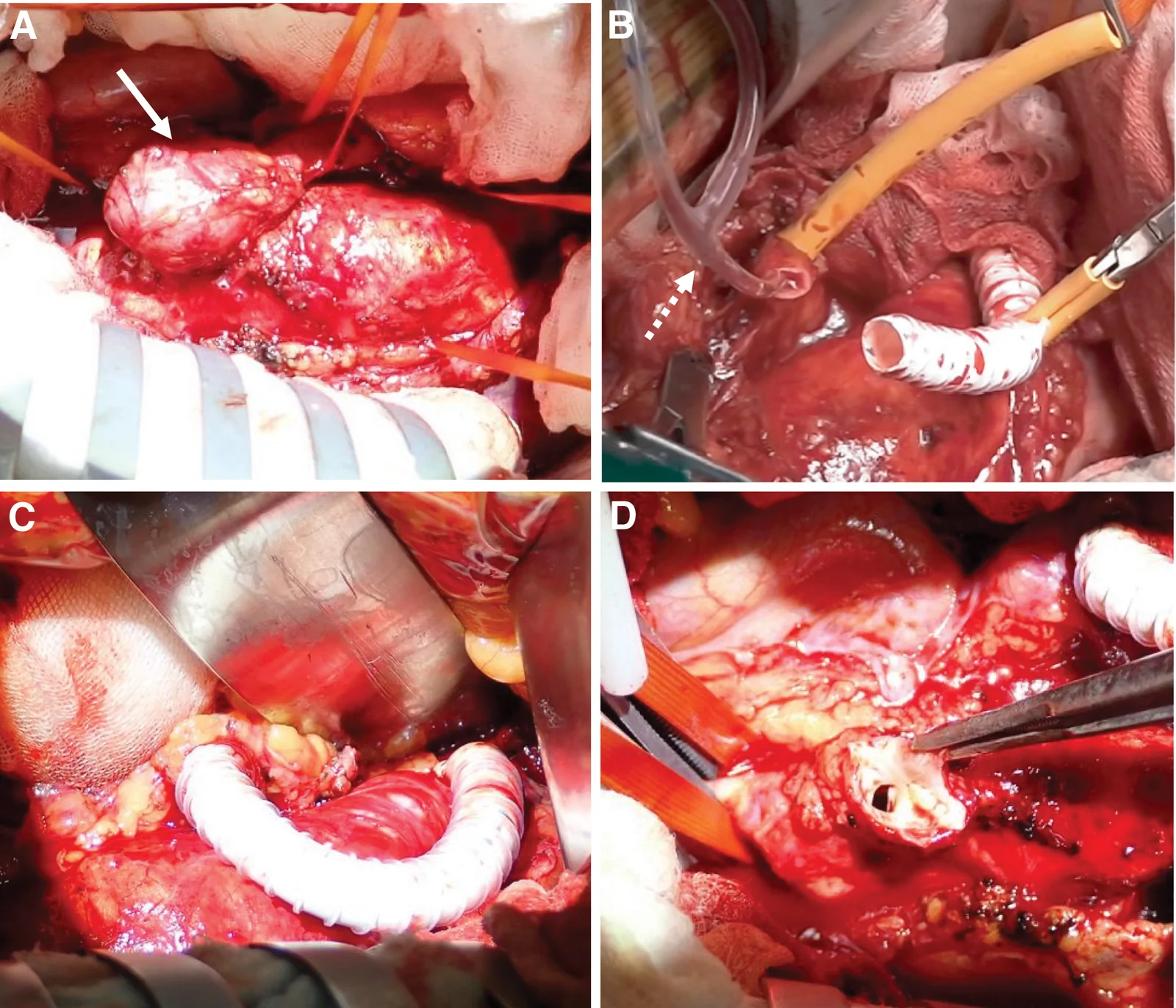
Intraoperative findings. (**A**) Markedly dilated IMA aneurysm (arrow). (**B**) Temporary shunt perfusion to the IMA established through a sheath placed in the right femoral artery (dotted arrow). (**C**) Left EIA-to-IMA bypass using an 8-mm ePTFE graft. (**D**) IMA ostium after resection of the IMA aneurysm. EIA, external iliac artery; ePTFE, expanded polytetrafluoroethylene; IMA, inferior mesenteric artery

**Fig. 3:**
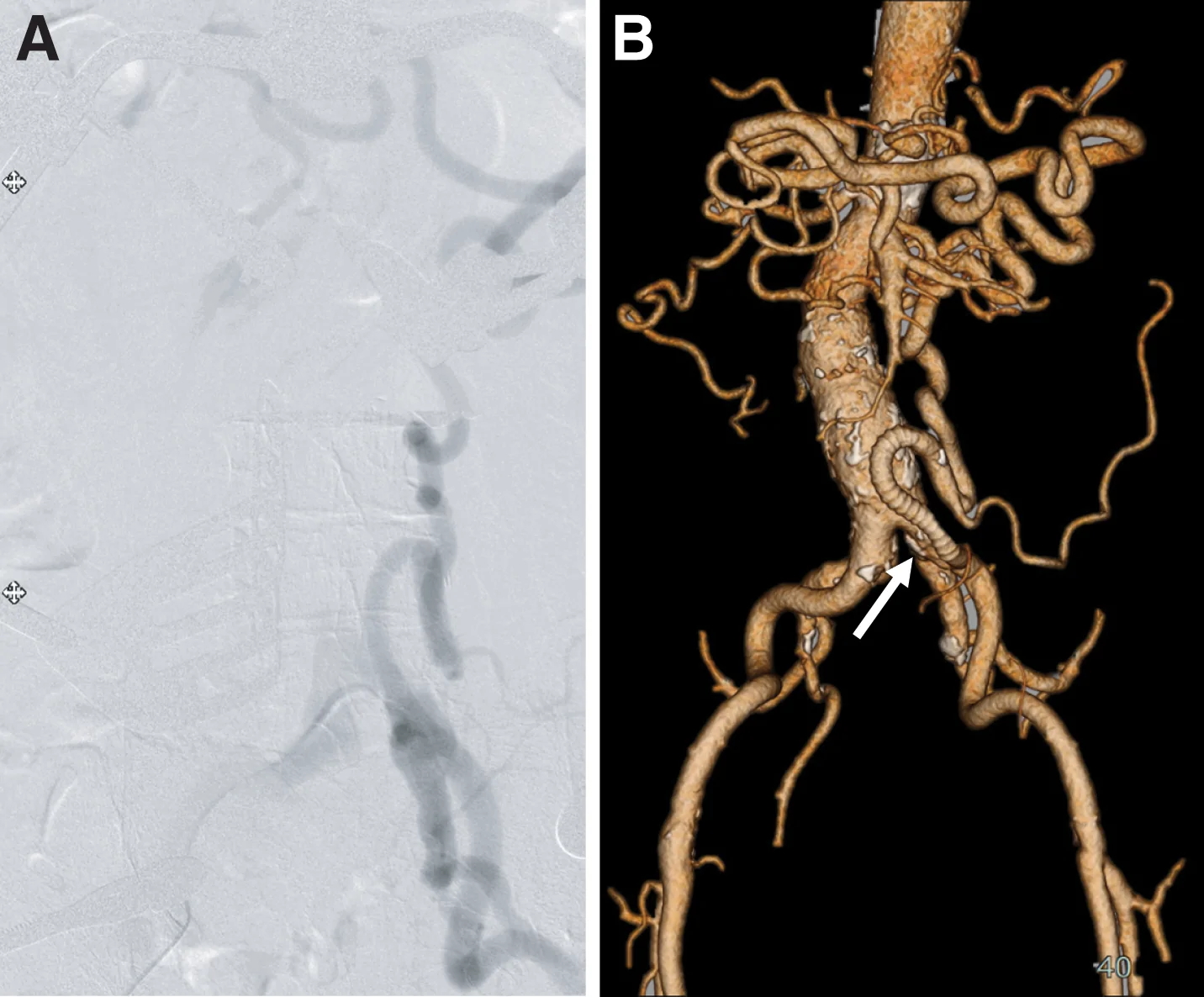
(**A**) DSA confirms bypass graft patency and adequate perfusion of the CA and SMA territories through the arc of Riolan. (**B**) Postoperative CT demonstrates patency of the left EIA-to-IMA bypass graft and perfusion of the mesenteric circulation through the IMA branches and dilated marginal arteries (arrow). CA, celiac artery; EIA, external iliac artery; IMA, inferior mesenteric artery; SMA, superior mesenteric artery

Initially, total arch replacement with concomitant aortic valve replacement was considered. However, to minimize the risk of mesenteric ischemia during circulatory arrest, a staged approach was adopted. Debranching thoracic endovascular aortic repair (TEVAR) was performed first (**Fig. 1B**). Fourteen days later, open repair of the IMA aneurysm was performed through a midline laparotomy using a transperitoneal approach. Intraoperatively, a markedly dilated IMA aneurysm and tortuous marginal arteries were identified (**Fig. 2A**). To minimize intestinal ischemia, a 6-Fr sheath was placed in the right femoral artery under US guidance, and temporary shunt perfusion to the IMA was established and maintained during bypass construction (**Fig. 2B**). Although shunt blood flow was not quantitatively measured, the anastomosis procedure was initiated after confirming the absence of adverse changes in hemodynamics or lactate levels. Before clamping the left external iliac artery (LEIA), 4000 IU of unfractionated heparin was administered, increasing the activated clotting time to approximately 250 seconds. A bypass from the LEIA to the IMA was then constructed using an 8-mm ring-reinforced expanded polytetrafluoroethylene (ePTFE) graft (**Fig. 2C**). The bypass graft flow was 560 ml/min. To prevent graft kinking caused by the compliance mismatch between the prosthetic graft and the native vessel, dissection of the distal IMA was minimized, the ringed graft length and course were carefully adjusted, and then the graft was subsequently secured within the retroperitoneum. After complete aneurysm resection, the IMA ostium was closed with felt-reinforced sutures (**Fig. 2D**). The abdominal aorta was then clamped to permit secure closure of the IMA ostium to prevent an anastomotic pseudoaneurysm or rupture at the closure site; the aortic clamp time was 15 minutes. After completion of all vascular procedures, protamine was administered to reverse the heparin effect. The retroperitoneum was closed to separate the graft from the intestinal tract. Completion angiography confirmed bypass graft patency and adequate perfusion of the CA and SMA territories through the arc of Riolan (**Fig. 3A**). Postoperative CT confirmed graft patency (**Fig. 3B**). Aspirin (100 mg/day) was initiated on POD 2. The patient was discharged without complications on POD 8 and remained asymptomatic at the 1-year follow-up.

## DISCUSSION

IMA aneurysms are the rarest form of visceral artery aneurysms, accounting for less than 1% of all cases. The reported distribution of splanchnic artery aneurysms is as follows: splenic artery (60%), hepatic artery (20%), SMA (6%), CA (4%), gastric and gastroepiploic arteries (4%), jejunal, ileal, and colic arteries (3%), pancreaticoduodenal and pancreatic arteries (2%), gastroduodenal artery (1.5%), and IMA (<1%).^7)^ Most IMA aneurysms arise from the proximal IMA trunk, which is exposed to substantial hemodynamic stress in patients with chronic mesenteric occlusive disease.

In patients with concomitant CA and SMA occlusion, the IMA often becomes the dominant collateral pathway supplying the mesenteric circulation through the arc of Riolan. This chronic high-flow state may lead to progressive arterial remodeling and aneurysmal degeneration, a mechanism referred to as the “jet disorder phenomenon.”^5,8,9)^ Similar mechanisms have been described in patients with aortoiliac occlusive disease.^10)^ In the present case, extensive collateralization between the IMA and SMA, together with markedly enlarged and tortuous marginal arteries, strongly supported this pathophysiological mechanism.

To the best of our knowledge, no definitive size threshold has been established for intervention in IMA aneurysms. However, rupture has been reported in aneurysms as small as 1 cm, suggesting that repair should be considered for most IMA aneurysms regardless of size.^11–14)^ Reported treatment strategies include aneurysm resection alone, IMA reimplantation, SMA bypass, combined IMA and SMA revascularization, and endovascular approaches such as chimney grafting and coil embolization.^13,15–22)^

The most important clinical issue in the present case was selection of the optimal revascularization strategy. SMA revascularization has been advocated to reduce the risk of mesenteric ischemia, decrease excessive collateral flow through the IMA, and potentially prevent recurrent aneurysmal degeneration.^13,14,18,23,24)^ Nevertheless, the optimal strategy remains controversial because of the rarity of this condition and the lack of long-term evidence.

Although SMA revascularization was carefully considered in the present case, several anatomical factors made this approach less suitable. First, severe circumferential calcification completely obstructed the SMA origin, making endovascular recanalization infeasible. Second, antegrade bypass from the supraceliac aorta would have substantially increased operative invasiveness and complexity. Third, retrograde SMA bypass from the iliac artery would have required extensive mesenteric dissection and prolonged operative time. In contrast, collateral perfusion through the arc of Riolan was already well established. Therefore, isolated IMA revascularization was considered sufficient to preserve mesenteric perfusion while minimizing operative risk.

Another important consideration is the long-term durability of this strategy. Persistent CA and SMA occlusion may continue to impose hemodynamic stress on the mesenteric collateral network even after surgery. Therefore, long-term surveillance using contrast-enhanced CT at 6 months, 12 months, and annually thereafter is mandatory to detect recurrent aneurysmal degeneration or graft-related complications.

Finally, graft selection and routing should be considered. Although Dacron and autologous vein grafts have been used previously,^10,13)^ we selected a ring-reinforced ePTFE graft because preoperative vein mapping demonstrated a size mismatch between the great saphenous vein and the IMA.

This case highlights that strategic isolated IMA revascularization may be a reasonable alternative when SMA revascularization is anatomically unsuitable in patients with complex mesenteric occlusive disease.

## CONCLUSIONS

We successfully treated an IMA aneurysm associated with CTO of the CA and SMA using isolated IMA revascularization and aneurysm resection. Strategic isolated IMA revascularization may be a feasible alternative when SMA revascularization is anatomically unsuitable. Long-term imaging surveillance remains essential because persistent mesenteric occlusive disease may predispose patients to recurrent aneurysmal degeneration.

## DECLARATIONS

### Availability of data and materials

All data supporting the conclusions of this article are included in the manuscript.
